# Continued improvement in disease manifestations of acid sphingomyelinase deficiency for adults with up to 2 years of olipudase alfa treatment: open-label extension of the ASCEND trial

**DOI:** 10.1186/s13023-023-02983-0

**Published:** 2023-12-02

**Authors:** Melissa P. Wasserstein, Robin Lachmann, Carla Hollak, Antonio Barbato, Renata C. Gallagher, Roberto Giugliani, Norberto Bernardo Guelbert, Julia B. Hennermann, Takayuki Ikezoe, Olivier Lidove, Paulina Mabe, Eugen Mengel, Maurizio Scarpa, Ebubekir Senates, Michel Tchan, Jesus Villarrubia, Beth L. Thurberg, Abhimanyu Yarramaneni, Nicole M. Armstrong, Yong Kim, Monica Kumar

**Affiliations:** 1 Children’s Hospital at Montefiore and the Albert Einstein College of Medicine, 3411 Wayne Ave, 9th Floor, Bronx, NY 10467 USA; 2 Charles Dent Metabolic Unit, National Hospital for Neurology and Neurosurgery, London, UK; 3 Department of Endocrinology and Metabolism, Amsterdam UMC, University of Amsterdam, Amsterdam, The Netherlands; 4 Department of Clinical Medicine and Surgery, University of Naples “Federico II”, Naples, Italy; 5 Department of Pediatrics, The University of California San Francisco, San Francisco, CA USA; 6 Postgraduate Program in Genetics and Molecular Biology, Med Genet Serv & DR Brasil, HCPA, INAGEMP, DASA, and Casa Dos Raros, UFRGS, Porto Alegre, Brazil; 7 Reina Fabiola University Clinic, Córdoba, Argentina; 8 Villa Metabolica, Center for Pediatric and Adolescent Medicine, University Medical Center, Mainz, Germany; 9 Department of Hematology, Fukushima Medical University, Fukushima, Japan; 10 Department of Internal Medicine, La Croix St Simon Hospital, Paris, France; 11 Clinica Santa Maria, Santiago, Chile; 12 Clinical Science for LSD, SpinCS, Hochheim, Germany; 13 Regional Coordinator Centre for Rare Diseases, University Hospital of Udine, 33100 Udine, Italy; 14 Istanbul Medeniyet University, Istanbul, Turkey; 15 Department of Genetic Medicine, Westmead Hospital, Sydney, Australia; 16 Hematology Department, Hospital Universitario Ramón y Cajal, Madrid, Spain; 17 Sanofi, Cambridge, MA USA; 18 Sanofi, Bridgewater, NJ USA; 19 Sanofi, Paris, France

**Keywords:** Recombinant human acid sphingomyelinase, Dose escalation, Organomegaly, Lung diffusing capacity, Acid sphingomyelinase deficiency, Niemann–Pick type B, Niemann–Pick type A/B

## Abstract

**Background:**

Olipudase alfa is a recombinant human acid sphingomyelinase enzyme replacement therapy for non-central-nervous-system manifestations of acid sphingomyelinase deficiency (ASMD). The ASCEND randomized placebo-controlled trial in adults with ASMD demonstrated reductions in sphingomyelin storage, organomegaly, interstitial lung disease and impaired diffusion capacity of the lung (DL_CO_), during the first year of olipudase alfa treatment. In an ongoing open-label extension of the ASCEND trial, individuals in the placebo group crossed over to olipudase alfa, and those in the olipudase alfa group continued treatment.

**Results:**

Thirty-five of 36 participants continued in the extension trial, and 33 completed year 2. Change-from-baseline results are presented as least-square mean percent change ± SEM. Improvements in the cross-over group after 1 year of treatment paralleled those of the olipudase alfa group from the primary analysis, while clinical improvement continued for those receiving olipudase alfa for 2 years. In the cross-over group, percent-predicted DL_CO_ increased by 28.0 ± 6.2%, spleen volume decreased by 36.0 ± 3.0% and liver volume decreased by 30.7 ± 2.5%. For those with 2 years of olipudase alfa treatment, the percent predicted DL_CO_ increased by 28.5 ± 6.2%, spleen volume decreased by 47.0 ± 2.7%, and liver volume decreased by 33.4 ± 2.2%. Lipid profiles and elevated liver transaminase levels improved or normalized by 1 year and remained stable through 2 years of treatment. Overall, 99% of treatment-emergent adverse events were mild or moderate, with one treatment-related serious adverse event (extrasystoles; previously documented cardiomyopathy). No individual discontinued due to an adverse event.

**Conclusion:**

Treatment with olipudase alfa is well tolerated and reduces manifestations of chronic ASMD with sustained efficacy.

*Trial registration* NCT02004691 registered 9 December 2013, https://clinicaltrials.gov/ct2/show/NCT02004691

**Supplementary Information:**

The online version contains supplementary material available at 10.1186/s13023-023-02983-0.

## Introduction

Acid sphingomyelinase deficiency (ASMD) is an autosomal recessive lysosomal storage disorder resulting from disease-causing variants in the *SMPD1* gene (EC3.1.4.12) encoding ASM [1]. A spectrum of disease phenotypes characterizes ASMD. ASMD type A (historically known as Niemann–Pick disease [NPD] type A) is associated with severe neurodegeneration and is uniformly fatal in early childhood [2, 3]. Chronic visceral and chronic neurovisceral phenotypes (ASMD type B [OMIM607616]; NPD type B and ASMD type A/B [NPD type A/B or intermediate phenotype], respectively) have onset from childhood to early adulthood [4, 5]. Olipudase alfa (Xenpozyme®) is a recombinant human ASM enzyme replacement therapy (ERT) approved for the treatment of the non-central nervous system (CNS) manifestations of ASMD in children and adults. Olipudase alfa infusions, initiated using a within-patient dose escalation regimen designed to debulk tissue sphingomyelin gradually [6, 7], are associated with clearance of sphingomyelin storage and improvements in multiple clinical manifestations [8–13].

Progressive multi-organ system involvement is the primary driver of disease burden among those with chronic ASMD phenotypes. Visceral manifestations of ASMD include splenomegaly, hypersplenism, interstitial lung disease (ILD) [5, 14–17] with impaired diffusion capacity of the lung (DL_CO_) [14, 17–19], hepatomegaly and chronic, fibrotic liver disease [20], dyslipidemia, osteopenia, and thrombocytopenia [5, 14–17, 21]. Advanced lung disease, lung infections, and liver failure are leading causes of morbidity and early mortality in adults with ASMD [22]. The 1-year results of the randomized placebo-controlled ASCEND trial in adults with ASMD demonstrated that olipudase alfa treatment reduced sphingomyelin storage and was associated with clinically significant improvements in percent-predicted DL_CO_, organomegaly, and other disease measures relative to placebo [10].

Thirty-five of 36 participants have continued in an ongoing open-label extension of the ASCEND trial, during which those in the former placebo group crossed over to olipudase alfa treatment. Here we report results representing 2 years of treatment for the original olipudase alfa group and 1 year of treatment for the former placebo group.

## Methods

### Study design and participants

As reported for the 1-year primary analysis period of the randomized, placebo-controlled, double-blind ASCEND study (NCT02004691/EudraCT 2015–000371-26) [10], adults with ASMD were enrolled from Argentina, Australia, Brazil, Chile, France, Germany, Italy, Japan, the Netherlands, Spain, Turkey, the United Kingdom, and the United States. Institutional Review Boards approved the protocol, and patients provided written informed consent.

Adults diagnosed with ASMD confirmed by enzymatic assay or genotyping were eligible for enrollment and were required to have DL_CO_ ≤ 70% of predicted normal value and spleen volume ≥ 6 multiples of normal (MN). ASMD types B and A/B were not differentiated. Exclusion criteria included platelet counts < 60 × 10^9^/L, alanine aminotransferase (ALT) or aspartate aminotransferase (AST) > 250 IU/L, or total bilirubin > 1.5 mg/dL at screening (except for patients with Gilbert syndrome).

### Olipudase alfa administration

Participants received olipudase alfa via intravenous infusion once every 2 weeks with a target maintenance dose of 3 mg/kg. During the primary analysis period, those randomly assigned to the olipudase alfa group continued in the open-label extension at the olipudase alfa maintenance dose. Individuals who crossed over to olipudase alfa from placebo underwent an olipudase alfa dose escalation scheme over approximately 14 weeks as previously described [10].

### Efficacy outcomes

Liver sphingomyelin content was quantified from liver biopsies by computer morphometry of high-resolution light microscopy images as previously described [10, 23–25].

Liver and spleen volumes were determined from abdominal magnetic resonance imaging (MRI) and expressed as MN, where normal liver and spleen volumes were assumed to be 2.5% and 0.2% of body weight, respectively [26]. Severe and moderate organomegaly were defined as > 2.5 and > 1.25 to ≤ 2.5MN, respectively, for hepatomegaly and > 15 and > 5 to ≤ 15MN, respectively, for splenomegaly [26].

Transaminase plasma concentrations were measured with an upper limit of normal (ULN) of 36 IU/L for ALT and 34 IU/L for AST. Platelet counts were monitored and thrombocytopenia was defined as a platelet count threshold of < 150 × 10^9^/L. Fasting plasma lipid profiles assessed dyslipidemia over time and the parameters and corresponding normal ranges are shown in Additional file 1: Table S1.

Percent predicted DL_CO_ adjusted for hemoglobin was calculated using standardized formulas [27, 28]. Percent predicted DL_CO_ values > 80% were considered normal without impairment, > 60–80% mild impairment, 40–60% moderate impairment, and < 40% severe impairment [29, 30].

High-resolution computed tomography (HRCT) lung images were scored subjectively for ground glass appearance, ILD, and reticulonodular density on a scale from 0 (no disease) to 3 (severe disease) as previously described [9, 10].

Disease biomarkers included plasma lyso-sphingomyelin levels measured by liquid chromatography-tandem mass spectrometry (LC/MS/MS) as previously described [24].

### Safety

Safety and tolerability assessments included physical examinations, cardiac evaluations, clinical laboratory testing, safety biomarker plasma levels, and reporting of adverse events and infusion-associated reactions (IARs, indicative of an inflammatory response characterized by symptoms such as pyrexia/nausea/vomiting/fatigue/pain and associated with increases in pro-inflammatory laboratory values such as high sensitivity C-reactive protein or ferritin). Immunogenicity was assessed using a validated enzyme-linked immunosorbent assay to screen for anti-drug antibodies (ADA) and to determine titers if present. Furthermore, as previously described [10], IgG anti-drug antibody-positive samples were assayed for neutralizing activity.

### Analyses

Demographic and disease characteristics were summarized using descriptive statistics. Changes from baseline are presented as the least-square (LS) mean ± standard error of the mean (SE) percent change from analysis of covariance (ANCOVA) except for HRCT data, which are the LS mean ANCOVA absolute changes from baseline. Absolute and percent changes from baseline are determined from baseline values.

## Results

### Participants

**Fig. 1:**
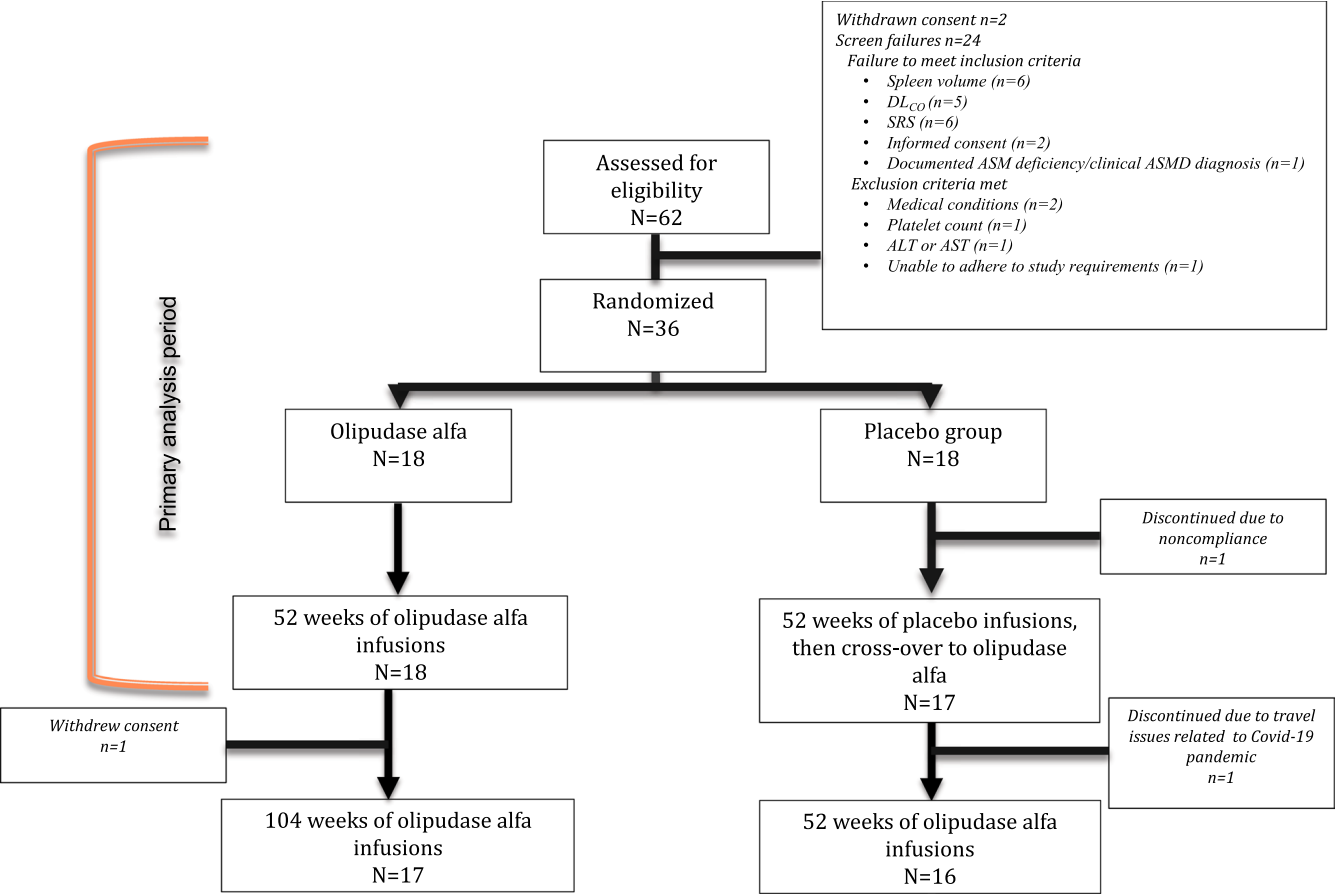
Patient disposition during the placebo-controlled and open-label extension periods of the ASCEND trial

Thirty-five individuals who completed the placebo-controlled primary analysis period of the ASCEND trial continued in the open-label trial extension, including 17 of 18 individuals crossing over to olipudase alfa from placebo and all 18 participants from the original olipudase alfa group (Fig. 1). Baseline characteristics at study screening are shown in Table 1.

**Table 1:** Demographics and baseline characteristics for all participants

	Overall (N = 36)
*Age (yr)*	
Mean (SD)	34.8 (14.9)
Median (min:max)	29.9 (18.6:65.9)
*Sex, n (%)*	
Female	22 (61)
Male	14 (39)
*Race, n (%)*	
Asian	2 (6)
White	32 (89)
Other	2 (6)
*Ethnicity, n (%)*	
Hispanic or Latino	11 (31)
Not Hispanic or Latino	24 (67)
Not Reported	1 (3)
*Age at ASMD diagnosis (yr)*	
Mean (SD)	18.0 (18.4)
Median (min:max)	6.4 (1:58)
*Years since ASMD diagnosis*	
Mean (SD)	16.8 (13.5)
Median (min:max)	16.3 (0:51)
*ASM activity (peripheral leukocytes), nmol/h/mg*	
Mean (SD)	0.119 (0.079)
Median (min:max)	0.10 (0:0.30)
*SMPD1 genotype, n (%)*	
Homozygous forArg610del	5 (13.9)
Heterozygous for Arg610del	10 (27.8)
Other variants	21 (58.3)

Thirty-three of 35 participants completed year 2 of the open-label extension period. One participant withdrew due to COVID-19 travel restrictions, and another withdrew consent (Fig. 1). Furthermore, some individuals had missing efficacy evaluations during the extension period due to the COVID-19 pandemic and prolonged restriction of access to pulmonary function testing and radiological assessments (Note: there was no impact of the COVID-19 pandemic during the primary analysis period as the last visit was in October 2019).

### Olipudase alfa treatment

Sixteen of the 17 individuals who crossed over to olipudase alfa from placebo reached the 3 mg/kg target dose of olipudase alfa during year 2, while one maintained a maximum dose of 2 mg/kg due to multiple missed infusions resulting from adverse events unrelated to study drug. All those from the initial olipudase alfa group received 3 mg/kg olipudase alfa dose during year 2. Some participants had periodic dose reductions or interruptions, sometimes requiring restarting the dose escalation. Among those completing year 2, six participants missed four or more infusions during year 2. The missed infusions resulted from COVID-19 pandemic issues for four of the six individuals. The mean ± SD treatment compliance during the extension period for those not impacted and those impacted by the COVID-19 pandemic was 92.7 ± 9.0% and 87.7 ± 14.9%, respectively. Data are not reported separately for individuals with missed infusions.

### Efficacy analyses

#### Sphingomyelin accumulation in liver

**Fig. 2:**
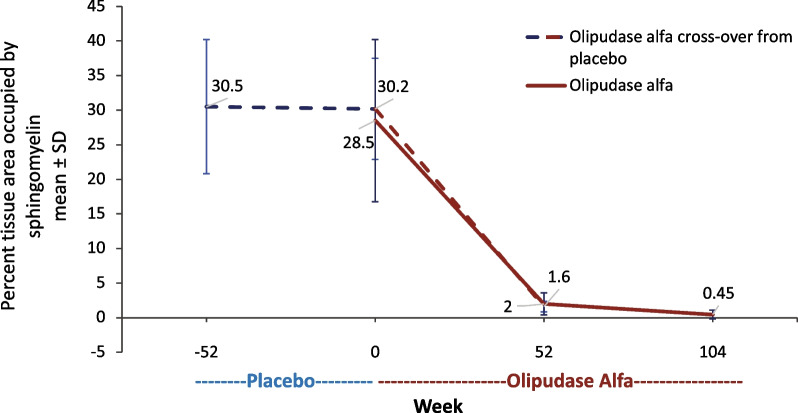
Sphingomyelin burden in liver tissue. The percent liver tissue area occupied by sphingomyelin is shown throughout the study during placebo or olipudase alfa treatment periods. Means are shown above and below the lines for the group crossing over to olipudase alfa from placebo and the olipudase alfa group, respectively. Liver tissue was obtained from biopsies at baseline, week 52, and week 104. Liver sphingomyelin burden was assessed by computer morphometry of high-resolution light microscopy imaging of stained tissue

Computer morphometry of high-resolution light microscopy images determined the mean ± SD percent tissue area occupied by sphingomyelin at baseline to be 28.5 ± 11.7% and 30.5 ± 9.7% in the original olipudase alfa and placebo groups, respectively (Fig. 2). Sphingomyelin clearance in liver biopsy samples was nearly complete after 1 year of olipudase alfa treatment within the former placebo group (mean ± SD percent tissue area occupied by sphingomyelin 1.6 ± 0.8%; LS mean ± SE percent change from baseline − 93.3 ± 5.0%). Within the group receiving olipudase alfa treatment for 2 years, the LS mean ± SE percent change from baseline was − 98.4 ± 2.0% (mean ± SD percent tissue area occupied by sphingomyelin 0.45 ± 0.6% at year 2).

#### Hepatomegaly

**Fig. 3:**
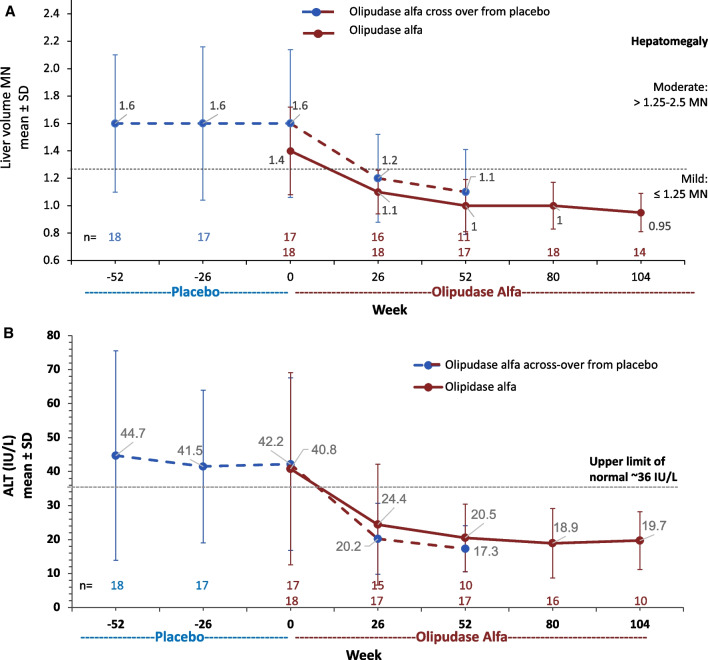
Liver volume in multiples of normal (MN) and alanine aminotransferase (ALT) during placebo or olipudase alfa treatment periods. **A** Liver volume over time. Means are shown above and below the lines for the group crossing over to olipudase alfa from placebo and the olipudase alfa group, respectively. The dashed lines indicate hepatomegaly severity cutoffs [26]. **B** ALT levels over time. Means are shown above and below the lines for the group crossing over to olipudase alfa from placebo and the olipudase alfa group, respectively. The dashed line indicates the upper limit of normal

Mean liver volume at baseline indicated moderate hepatomegaly, which improved with olipudase alfa treatment (Fig. 3A). Within the former placebo group, the LS mean ± SE percent change from baseline was -30.7 ± 2.5% after 1 year of olipudase alfa treatment (mean ± SD 1.6 ± 0.5 MN at baseline to 1.1 ± 0.3 MN at 1 year) while the decrease in liver volume for those receiving olipudase alfa for 2 years (mean ± SD 1.4 ± 0.3MN at baseline and 0.95 ± 0.1 MN at year 2; LS mean ± SE percent change from baseline − 33.4 ± 2.2%) was slightly improved relative to year 1 (LS mean ± SE percent change from baseline -27.8 ± 2.5%) [10].

Individual liver volumes over time are shown in Supplemental Material Fig. 1A. Liver volumes improved with olipudase alfa treatment in all individuals, with most values in the mild category by 1 year of olipudase alfa treatment among those whose baseline values were moderate (18/23). One individual with severe hepatomegaly at baseline was in the moderate category after 1 year of treatment, with a decrease from baseline of 40% (3 MN to 1.8 MN).

#### Lipid profiles

Mean baseline levels for antiatherogenic and proatherogenic lipids and lipoproteins were below and above normal limits, respectively [10] (Additional file 1: Table S1). Additional file 1: Table S1 lists the mean observed values and percent changes from baseline over time. Within the former placebo group, high-density lipoprotein cholesterol (HDL-C) and low-density lipoprotein cholesterol (LDL-C) LS mean ± SE percent changes from baseline were 59.8 ± 9.7% and − 27.5 ± 6.8%, respectively, after 1 year of treatment. Within the group continuing to receive olipudase alfa, HDL-C and LDL-C LS mean ± SE percent change from baseline were 64.4 ± 10.5% and − 23.0 ± 7.1%, respectively, after 2 years of treatment. Results were similar for other plasma lipoproteins and lipids, where levels of proatherogenic parameters (total cholesterol, triglycerides, apolipoprotein B, very low-density lipoprotein cholesterol, non-HDL-C) decreased, and levels of the antiatherogenic parameter apolipoprotein A1 increased (Additional file 1: Table S1).

#### Pre-infusion plasma transaminase levels

Mean ± SD transaminase levels at baseline were elevated (ALT: 44.7 ± 30.8 for the placebo group and 40.8 ± 28.3 IU/L for the olipudase alfa group (ULN 36 IU/L), reflecting increased levels for 50% of individuals (18/36 with levels > 36 IU/L). Levels improved over time with olipudase alfa treatment. The mean ± SD ALT after 1 year of olipudase alfa treatment for the former placebo group was 17.3 ± 6.8 (LS mean ± SE percent change from baseline − 45.2 ± 9.1%) and was 19.7 ± 8.5 IU/L (LS mean ± SE percent change from baseline − 32.0 ± 10.2%) after 2 years of olipudase alfa (Fig. 3B), and only one individual had an ALT level > 36 IU/L. Results were similar for AST (data not shown).

#### Splenomegaly

**Fig. 4:**
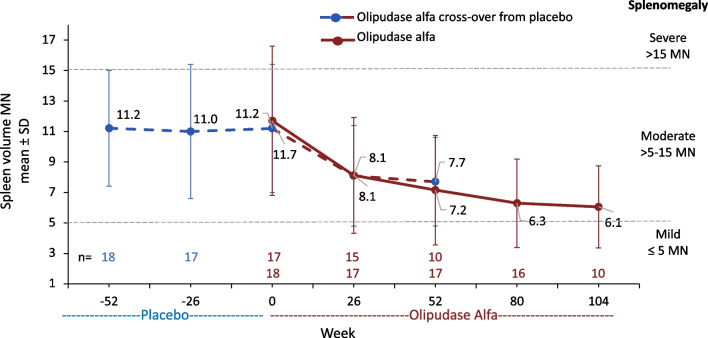
Spleen volume in multiples of normal (MN) during placebo or olipudase alfa treatment periods. Means are shown above and below the lines for the group crossing over to olipudase alfa from placebo and the olipudase alfa group, respectively. Splenomegaly severity cutoffs are indicated by the dashed lines [26]

Mean baseline spleen volume reflected moderate to severe splenomegaly and decreased with olipudase alfa treatment (Fig. 4). Within the former placebo group, the LS mean ± SE percent change from baseline was − 35.9 ± 3.0% with 1 year of olipudase alfa treatment (mean ± SD spleen volume 11.2 ± 3.8 MN at baseline versus 7.7 ± 2.9 MN at 1 year). The LS mean percent change from baseline was − 47.0 ± 2.7% for the group receiving olipudase alfa for 2 years (11.7 ± 4.9 MN at baseline versus 6.1 ± 2.7MN at year 2) and reflected further improvement compared to year 1 (LS mean percent change from baseline − 39.5 ± 2.4%) [10].

Individual spleen volumes over time are shown in Supplemental Material Fig. 1B. Spleen volumes improved with olipudase alfa treatment in all individuals by 6 months of olipudase alfa treatment. At their last assessment while receiving olipudase alfa, eight individuals improved from severe to moderate splenomegaly, and 11 improved from moderate to mild.

#### Thrombocytopenia

Mean baseline platelet counts reflected mild thrombocytopenia with a broad range of values from 63.6 to 207 × 10^9^/L [10]. Within the former placebo group, mean ± SD platelet counts increased from 115.6 ± 36.3 × 10^9^/L at baseline to 140.0 ± 50.8 × 10^9^/L after 1 year of olipudase alfa (range 76–245 × 10^9^/L; LS mean ± SE percent change from baseline 21.7 ± 6.4%). Among those with 2 years of olipudase alfa treatment, platelet counts increased from a mean ± SD of 107.2 ± 26.9 × 10^9^/L at baseline to 133.6 ± 29.6 × 10^9^/L at year 2 (range 95–199.5 × 10^9^/L; LS mean ± SE percent change from baseline 24.9 ± 6.9%).

#### Pulmonary endpoints

**Fig. 5:**
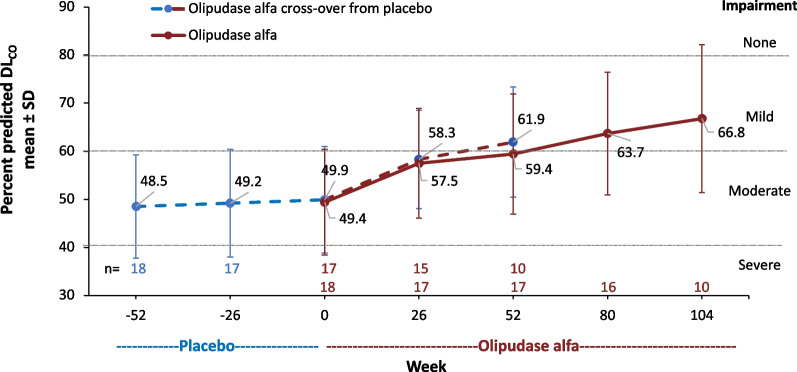
Percent predicted diffusing  capacity of the lung for carbon monoxide (DL_CO_) adjusted for hemoglobin and barometric pressure during placebo or olipudase alfa treatment periods. Means are shown above and below the lines for the group crossing over to olipudase alfa from placebo and the olipudase alfa group, respectively. Percent predicted DL_CO_ values > 80% were considered normal without impairment, > 60–80% mild impairment, 40–60% moderate impairment, and < 40% severe impairment [29, 30]

Figure 5 shows the mean percent predicted DL_CO_ over time. The mean baseline percent predicted DL_CO_ indicated moderate impairment of the diffusing capacity of the lung [10]. Within the former placebo group, the mean ± SD percent predicted DL_CO_ improved with 1 year of olipudase alfa treatment from 48.5 ± 10.8% at baseline to 61.9 ± 11.4% at year 1 (LS mean ± SE percent change from baseline 28.0 ± 6.2%). Within the group with 2 years of olipudase alfa treatment, the mean ± SD percent predicted DL_CO_ improved from 49.4 ± 11.0% at baseline to 66.8 ± 15.4% at year 2 (LS mean ± SE percent change from baseline 28.5 ± 6.2%) and reflected continued improvement from the increase reported at year 1 (LS mean ± SE percent change from baseline 22.2 ± 3.4%) [10].

Individual percent predicted DL_CO_ values over time are shown in Supplemental Material Fig. 1C. Among 10 individuals with 2 years of olipudase alfa treatment, impairment improved to mild or none for all but one individual with the most severe value at baseline. Individuals from the placebo group receiving olipudase alfa for 6 months to 1 year showed improved percent predicted DL_CO_ over time.

**Fig. 6:**
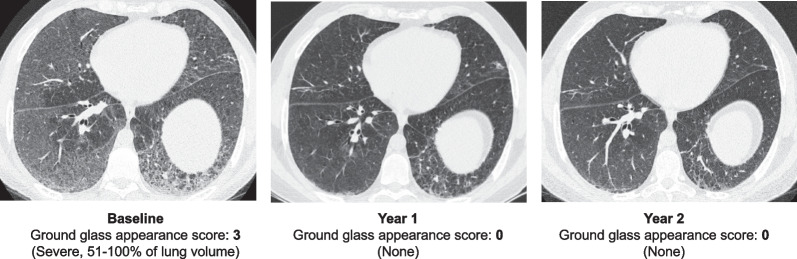
Lung high-resolution computed tomography (HRCT). Illustrative high-resolution computerized tomography image of ground glass opacity, reflecting sphingomyelin-filled infiltrates at baseline and after 1 or 2 years of olipudase alfa treatment

The mean lung imaging HRCT scores at baseline for ground glass appearance reflected mild ILD [10], and mean scores improved (decreased) over time with olipudase alfa treatment (Table 2). An illustrative HRCT image from an individual with 2 years of olipudase alfa treatment (Fig. 6) shows clearance of ground glass opacities at year 1 that is maintained at year 2.

**Table 2:** Lung high-resolution computed tomography (HRCT) scores for ground glass appearance

	Cross-over from placebo group*			Olipudase alfa group**	
	Baseline n = 18	Olipudase alfa for 1 year n = 14	Baseline n = 18	Year 1 n = 18	Year 2 n = 16
Mean ± SD	0.53 ± 0.64	0.22 ± 0.35	0.65 ± 0.72	0.15 ± 0.29	0.13 ± 0.25
LS mean ± SE change from baseline		− 0.37 ± 0.08		− 0.45 ± 0.13	− 0.48 ± 0.07

*Received olipudase alfa for 1 year

^**^Original olipudase alfa group received olipudase alfa for 2 years

*LS* least square, *SE* standard error of the mean

#### Biochemical marker levels

**Fig. 7:**
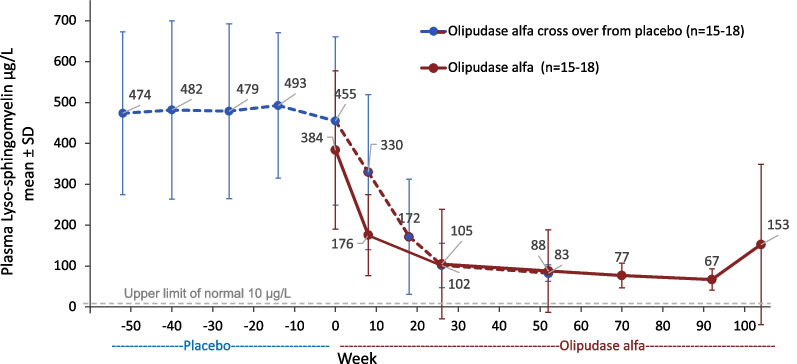
Pre-infusion plasma lysosphingomyelin levels during placebo or olipudase alfa treatment periods. The dashed line indicates the upper limit of normal. (*Note*: The increase in mean level at week 104 resulted from multiple missed infusions by two participants.)

Plasma levels of the sphingomyelin metabolite lyso-sphingomyelin were elevated at baseline in all participants (mean ± SD: 474 ± 199 μg/L in the placebo group and 384 ± 194 μg/L in the olipudase alfa group; overall range 157–830 μg/L; ULN 10 μg/L). Pre-infusion levels steadily decreased and stabilized after 6 months of olipudase alfa treatment (Fig. 7). The increase in the mean level observed at week 104 resulted from multiple missed olipudase alfa infusions by two participants within the group.

### Treatment-emergent adverse events and other safety assessments

The treatment-emergent adverse event profile during olipudase alfa treatment is summarized in Table 3. Most events were mild or moderate in severity (572/584, 97.9%). No event led to permanent treatment discontinuation or study withdrawal. Among 13 serious events reported for ten participants, one event of extrasystoles in an individual with previously documented cardiomyopathy was considered possibly treatment-related. There were 151 events considered possibly related to treatment (Table 3). The most common related adverse event was headache among 10/35 individuals (28.6%), and most were categorized as IARs (53/59 events; Table 3). Among the 101 events classified as IARs, most (63/101, 62%) were reported in the first 6 months of treatment, and none were reported after 18 months. Four individuals had multiple transient increases in liver transaminases, and all resolved.

**Table 3:** Overview of treatment-emergent adverse events during treatment with olipudase alfa (1 year of treatment for those in the former placebo group crossing over to olipudase alfa and 2 years of treatment for the original olipudase alfa group)

Adverse event	All olipudase alfa-treated (N = 35)	
	No. individuals (%)	No. Events (%)
Any treatment-emergent adverse event	35 (100)	584
Severity		
Mild	34 (97.1)	447 (76.5)
Moderate	26 (74.3)	125 (25.9)
Severe	6 (17.1)	11 (1.9)
Any serious treatment-emergent adverse events	10 (28.6)	13 (2.2)
Any treatment-emergent adverse events potentially related to study drug	24 (68.6)	151 (25.9)
Any serious treatment-emergent adverse events potentially related to study drug	1 (2.9)	1 (0.2)

Related adverse events reported in two or more individuals	No. individuals (%)	No. events	Events categorized as IARs	Severity
Headache	10 (28.6)	59	53/59	47 mild, 11 moderate
Alanine aminotransferase increased	4 (11.4)	11	1/11	6 mild, 4 moderate, 1 severe
Aspartate aminotransferase increased	4 (11.4)	6	1/6	3 mild, 3 moderate
Urticaria	3 (8.6)	5	5/5	3 mild, 2 moderate
Vomiting	3 (8.6)	4	4/4	All mild
Abdominal pain	3 (8.6)	3	1/3	2 mild, 1 moderate
Nausea	3 (8.6)	3	3/3	2 mild, 1 moderate
Pyrexia	3 (8.6)	3	2/3	2 mild, 1 moderate
Pruritis	2 (5.7)	2	2/2	1 mild, 1 moderate
Joint swelling	2 (5.7)	5	4/5	All mild
Chills	2 (5.7)	2	0/2	All mild
Blood bilirubin increased	2 (5.7)	2	0/2	All mild
Dyspnea	2 (5.7)	2	1/2	All mild
Erythema	2 (5.7)	2	2/2	All mild
Musculoskeletal chest pain	2 (5.7)	2	0/2	All mild

*IAR* infusion associated reaction

No individual developed neutralizing anti-drug IgG antibodies that interfered with ASM enzyme cellular uptake through 2 years of olipudase alfa treatment. One individual had transient positivity for neutralizing antibodies that inhibited ASM catalytic activity after 1 year of olipudase alfa treatment without apparent impact on clinical responses.

## Discussion

Olipudase alfa is the first disease-modifying treatment for the non-CNS manifestations of ASMD in children and adults. The primary analysis period of the ASCEND clinical trial assessed olipudase alfa versus placebo in adult patients with chronic ASMD for 1 year [10]. Treatment with olipudase alfa cleared tissue sphingomyelin levels, reduced plasma levels of lyso-sphingomyelin, and produced clinically relevant improvements in multiple ASMD manifestations compared to placebo [10]. Individuals who crossed over from the placebo group and received 1 year of olipudase alfa treatment in the extension study had improvements in liver, spleen, and lung endpoints comparable to those who received olipudase alfa for 1 year during the primary analysis period. While some individuals had missing efficacy evaluations during the extension period due to the COVID-19 pandemic, there was only one discontinuation due to COVID-19-related travel issues.

The improvements in clinical disease measures were consistent with those reported in other olipudase alfa trials in adults and children with chronic ASMD [8, 9, 11–13]. Adults receiving 2 years of olipudase alfa treatment had continued improvement and, in some cases, normalization of clinical manifestations over time, which has also been demonstrated in the pediatric population after 2 years of treatment [12] and for five adults receiving olipudase alfa for 6.5 years [13]. Overall, individuals had improvements in clinical measures of ASMD irrespective of baseline severity, as shown by individual responses, and improvements persisted or continued in the second year of treatment. Olipudase alfa was well-tolerated. Most adverse events were non-serious, non-treatment-related, and mild in severity through 2 years of treatment. ASMD is panethnic, thus it is important to note that the population of the ASCEND trial was predominantly Caucasian and the participants were primarily from United States, Europe, or South America.

Based on natural history study data for untreated individuals, the multi-organ manifestations of chronic ASMD worsen over time or fail to improve [15, 17]. Progressive liver disease, cirrhosis, and liver failure are the major contributors to morbidity and mortality in patients with chronic ASMD [22]. Olipudase alfa treatment resulted in almost complete clearance of sphingomyelin storage from the liver after 2 years. This clearance was paralleled by the normalization of liver volume and biochemical assessments of liver function. Dyslipidemia, known to worsen over time in chronic ASMD [5, 17], improved with continued olipudase alfa treatment, particularly for levels of anti-atherogenic lipids, as has been reported previously [24].

Olipudase alfa treatment reduced splenomegaly, which continued to improve after 1 year. Reduction in spleen volume was accompanied by increased platelet counts indicating correction of secondary hypersplenism that contributes to thrombocytopenia [31].

Lung disease and respiratory complications, such as infections, worsen with age among those with chronic ASMD [5] and are significant contributors to mortality [17, 22]. No serious treatment-emergent adverse events of pneumonia were reported during the extension period. Olipudase alfa infusions for 2 years were associated with continued improvement in lung diffusing capacity and were accompanied by resolution of “ground glass” opacities indicating elimination of cellular infiltrates [32]. While the COVID-19 pandemic impacted the completion of some pulmonary assessments, over half of the participants had pulmonary data at 2 years.

## Conclusions

In summary, this open-label extension study of olipudase alfa in adults with ASMD extends the findings of the randomized, double-blind, placebo-controlled trial. The study demonstrates that olipudase alfa infusions remain well-tolerated and effective. Many of the clinical features of ASMD normalized after 2 years of treatment, and monitoring continues in the extension study to evaluate the stability of the results and additional improvements. The improvements in visceral ASMD disease with olipudase alfa treatment will significantly impact the disease burden for those with this progressive multi-organ disorder.

### Supplementary Information

**Additional file 1: Supplemental Figure 1.** Individual responses over time for liver volumes (**A**), spleen volumes (**B**), and derived % predicted DLCO adjusted for hemoglobin and pressure (**C**), and **Supplementary Table 1.** Observed values and percent change from baseline for fasting plasma lipoprotein and lipid levels.

## Data Availability

Qualified researchers may request access to patient-level data and related study documents, including the clinical study report, study protocol with any amendments, blank case report form, statistical analysis plan, and dataset specifications. Patient-level data will be anonymized, and study documents redacted to protect the privacy of trial participants. Further details on Sanofi's data sharing criteria, eligible studies, and process for requesting access can be found at: https://vivli.org.
