# Supplementary material for: Continued improvement in disease manifestations of acid sphingomyelinase deficiency for adults with up to 2 years of olipudase alfa treatment: open-label extension of the ASCEND trial
Source: Orphanet J Rare Dis. 2023 Dec 2;18:378. doi: 10.1186/s13023-023-02983-0 (PMC10693698; doi:10.1186/s13023-023-02983-0)
Supplement: Supplementary file 1 — Additional file 1: Supplemental Figure 1. Individual responses over time for liver volumes (A), spleen volumes (B), and derived % predicted DLCO adjusted for hemoglobin and pressure (C), and Supplementary Table 1. Observed values and percent change from baseline for fasting plasma lipoprotein and lipid levels. [file 13023_2023_2983_MOESM1_ESM.docx]

**Supplemental Material**

**Supplemental Figure 1.** Individual responses over time for liver volumes (A), spleen volumes (B), and derived percent predicted DL_CO_ adjusted for hemoglobin and barometric pressure (C). The dotted lines indicate individuals originally in the placebo group who crossed over to olipudase alfa treatment at time 0.

A.


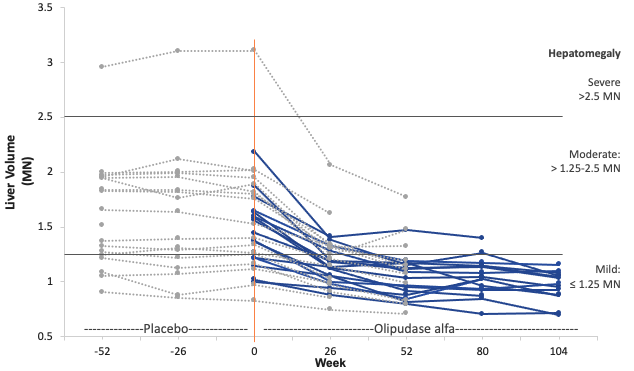


B.

C.


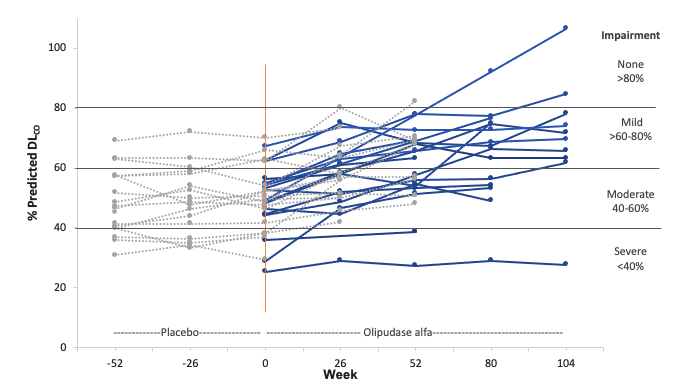


**Supplementary Table 1. Observed values and percent change from baseline for fasting plasma lipoprotein and lipid levels among those in ASCEND (N=36)**

|  | **Olipudase alfa after cross-over from placebo*** | | **Olipudase alfa**** | | |
| --- | --- | --- | --- | --- | --- |
| **Parameter**  *Normal values* | **Study Baseline**  **N=18** | **1 yr*****  **N=14** | **Study Baseline**  **N=18** | **1 yr**  **N=18** | **2 yr**  **N=12** |
| **Antiatherogenic** |  |  |  |  |  |
| **HDL-C mmol/L**  *>0.78 US male, >0.91 US female, >1.2 UK mmol/L* |  |  |  |  |  |
| Observed value  Mean (SD) | 0.534 (0.2529) | 0.795 (0.3293) | 0.617 (0.2186) | 0.816 (0.2439) | 1.000 (0.2900) |
| % Change from baseline  LS mean (SE) | - | 59.824 (9.7047) | - | 39.928 (6.7810) | 64.396 (10.5285) |
| **APO-A1 g/L**  *1.02 - 2.00 g/L* |  |  |  |  |  |
| Observed value  Mean (SD) | 0.825 (0.1838) | 0.959 (0.2390) | 0.846 (0.1949) | 0.956 (0.2178) | 1.063 (0.1642) |
| % Change from baseline  LS mean (SE) | - | 20.075 (5.1294) | - | 15.035 (3.7198) | 23.528 (5.5582) |
| **Proatherogenic** |  |  |  |  |  |
| **APO-B g/L**  *US male 0.55-1.4 g/L; US female 0.55 -1.25 g/L; UK 0.52-1.09 g/L* |  |  |  |  |  |
| Observed value  Mean (SD) | 1.463 (0.4452) | 0.960 (0.2483) | 1.251 (0.2888) | 0.877 (0.2290) | 0.882 (0.2694) |
| % Change from baseline  LS mean (SE) | - | -30.884 (4.0817) | - | -30.421 (3.8851) | -31.231 (4.4231) |
| **LDL-C mmol/L**  *US <3.3 mmol/L; UK 0-2 mmol/L* |  |  |  |  |  |
| Observed value  Mean (SD) | n=17  4.010 (1.6851) | 2.758 (0.9547) | 3.558 (0.7421) | 2.578 (0.7054) | 2.732 (1.0869) |
| % Change from baseline  LS mean (SE) | - | n=13  -27.543 (6.8190) | - | -25.743 (4.7913) | -22.995 (7.1007) |
| **VLDL-C mmol/L**  *US <0.518 mmol/L; UK 0.09-0.71 mmol/L* |  |  |  |  |  |
| Observed value  Mean (SD) | n=17  1.065 (0.3328) | 0.788 (0.4568) | 0.873 (0.3388) | 0.559 (0.1537) | 0.566 (0.2541) |
| % Change from baseline  LS mean (SE) | - | n=13  27.402 (9.0003) | - | -32.970 (5.1067) | -34.842 (9.3837) |
| **Total-C mmol/L**  *US <5.18 mmol/L; UK 0-3.9 mmol/L* |  |  |  |  |  |
| Observed value  Mean (SD) | 5.472 (1.2164) | n=15  4.356 (0.9556) | 4.749 (0.8762) | 3.949 (0.7717) | 4.293 (1.0380) |
| % Change from baseline  LS mean (SE) | - | n=15  -17.162 (4.4187) | - | -17.919 (3.8886) | -13.143 (4.9658) |
| **NonHDL-C mmol/L**  *3.4-4.0 mmol/L; <3.4 for those at risk of heart disease* |  |  |  |  |  |
| Observed value  Mean (SD) | 5.124 (1.7487) | 3.544 (1.0109) | 4.431 (0.9372) | 3.133 (0.7685) | 3.293 (1.1280) |
| % Change from baseline  LS mean (SE) | - | -25.839 (5.3285) | - | -29.822 (4.0064) | -26.777 (5.7693) |
| **TG mmol/L**  *<1.69 mmol/L* |  |  |  |  |  |
| Observed value  Mean (SD) | 2.497 (1.0051) | 1.719 (0.9958) | 1.908 (0.7423) | 1.216 (0.3363) | 1.237 (0.5589) |
| % Change from baseline  LS mean (SE) | - | -28.008 (8.5606) | - | -33.830 (5.4778) | -34.990 (9.2879) |

* received olipudase alfa for 1 year after cross-over

**original olipudase alfa group received olipudase alfa for 2 years

*** 1 year of olipudase alfa treatment following 1 year of placebo

APO-A1 apolipoprotein A1, APO-B apolipoprotein B, HDL-C high density lipoprotein cholesterol, LDL-C low density lipoprotein cholesterol, NonHDL-C non high density lipoprotein cholesterol, Total-C total cholesterol, TG triglyceride, VLDL-C very low density lipoprotein cholesterol
